# Community nurse resource implications for a change in heparin prophylaxis policy

**DOI:** 10.1051/sicotj/2015013

**Published:** 2015-06-05

**Authors:** Martyn J. Parker

**Affiliations:** 1 Orthopaedic Consultant, Peterborough and Stamford Hospital NHS Foundation Trust, Department of Orthopaedics, Peterborough City Hospital CBU PO Box 211 Core C, Bretton Gate Peterborough PE3 9GZ UK

**Keywords:** Hip fracture, Thrombo-prophylaxis, Heparin

## Abstract

*Introduction*: A review was undertaken for a consecutive series of hip fracture patients for the year before and then after a change in low dose heparin prophylaxis policy.

*Patients and methods*: For the first year heparin was administered in hospital for a maximum of 14 days only. Patients sent home before this time were not discharged taking heparin. For the second year heparin was administered as recommended by NICE guidelines for 28 days from admission regardless of whether the patient was discharged.

*Results*: For the first year 486 patients were treated with a mean of 10.4 doses of heparin per patient. For the second year 465 patients were treated with a mean of 24.3 doses per patient. In total an extra 6,464 doses of heparin were administered. 33.8% of patients were unable to administer their heparin at home therefore a district nurse administered 2,284 of these doses of subcutaneous heparin at the patient’s home. The increased cost associated with the change in policy was estimated to be £161 per patient, with over 90% of this increase being incurred by the district nurse expense. If applied nationally for the England, using extended heparin prophylaxis for hip fracture patients would cost in excess of 12 million pounds each year.

*Conclusion*: Whilst the necessity for and duration of thromboembolic prophylaxis for these patients remains undetermined, there is a need to re-evaluate the cost effectiveness of the current recommendations for hip fracture patients.

## Introduction

Continued controversy and debate exists about the role of pharmaceutical anti-thrombotic prophylaxis after major orthopaedic surgery [1, 2]. Conflicting and changing advice is provided by different guidelines [3–5]. This is due to different interpretations of the available evidence and the lack of large, independently conducted, randomised trials. Reported randomised trials to date demonstrate that heparin prophylaxis has no significant effect on mortality after hip fracture. Thromboembolic complications appear to be reduced but at the expense of an increased occurrence of bleeding complications [4–6].

The policy at our hospital was traditionally to give a short course of heparin for 14 days and not to discharge patients on heparin. More recent studies have suggested that a longer course of heparin should be used [7, 8]. The NICE guidelines on thromboembolic prophylaxis have recommended to offer to all hip fracture patients who have no contraindications, a 28- to 35-day course of subcutaneous low-molecular weight heparin [4].

On the 1st January 2013 a change in policy from the shorter course heparin, to a standard 28-day course for hip fracture patients was made at Peterborough City Hospital. The aim of this study was to review the effect of this modification in policy for the year before and after this change in heparin use, with particular reference to the use of district nurse resources.

## Patients and methods

Information was collected on admission and during the patients’ hospital stay as part of an ongoing audit project for all hip fracture patients admitted to a single district general hospital. At 6 weeks after discharge from hospital all surviving patients were reviewed in a hip fracture clinic. Any patients unable to attend were contacted by phone to enquire about any complications that had occurred since discharge from hospital. In addition for the second year of the study details of heparin administration at home were sought.

For the first year of study (1st January 2012 to 31 December 2012), the thromboembolic prophylaxis policy was to administer low-molecular weight heparin starting from admission for 14 days. No heparin was administered after discharge of the patient, even if the patient was discharged within 14 days of admission, so some patients received less than 14 days of heparin. The ward nursing staff administered all heparin. For the second time period of the study (1st January 2013 to 31 December 2013), heparin was administered for a total of 28 days from admission, regardless of whether the patient was discharged in accordance with the updated NICE guideline [4].

The cost of the heparin was supplied by the hospital pharmacy and the cost of a district nurse visit by a search of the literature [9]. Statistical analysis between year groups was performed with the Fisher Exact test.

## Results

For the year 1st January 2012 to 31st December 2012, 486 patients were treated with a mean of 10.4 doses of heparin per patient. For the year 1st January 2013 to 31st December 2013, 465 patients were admitted with a hip fracture and 307 patients who discharged on heparin (Table 1). For those patients discharged on heparin 105 agreed to self-administer their heparin themselves or for a relative to administer the heparin. One hundred and fifty-seven patients felt unable to or had no relative who could administer the heparin and therefore required a district nurse to visit daily to give the heparin after discharge from hospital. This included 40 patients who lived in residential homes where there was no nurse on site to give injections. The reason for the nine patients with incomplete information was seven patients had died before attending the clinic and two patients were lost to follow-up.

**Table 1.** Details of how patients completed their heparin course.

	Number (%)
*Heparin not indicated*	
Heparin contraindicated	21 (4.5%)
Patient on warfarin	27 (5.8%)
Patient on rivaroxaban	1 (0.2%)
Heparin not indicated as old fracture and patient mobile	1 (0.2%)
*Heparin completed in hospital*	
Heparin course completed before discharge	89 (19.1%)
Patient died in hospital before the 28 course completed	19 (4.1%)
*Patients requiring heparin after discharge to complete course*	
Heparin not prescribed	5 (1.1%)
Heparin not given by nursing home	2 (0.4%)
Heparin not given by district nurse	1 (0.2%)
Heparin prescribed at home and administered by the patient	72 (15.5%)
Heparin prescribed at home and administered by a relative	33 (7.1%)
Patient did not complete course heparin at home	3 (0.6%)
Patient discharged to a nursing home where staff administered heparin	25 (5.4%)
Heparin prescribed at home and administered by a district nurse	157 (33.8%)
*Missing information*	
Unknown if patient completed the course of heparin	9 (1.9%)
TOTAL	465

For those 157 patients who required a district nurse visit, the mean number of visits per patient was 14.5 (range 1–26). In total 2,284 district nurse visits were undertaken. The cost for a district nurse visit was estimated at £30 corrected to 2013 prices (9). The estimated total cost for these visits for the year was £68,520. The cost for a single dose of dalteparin at 2013 prices was 97 pence. Changing from the previous 14-day course to a 28-day course of heparin involved an extra 6,496 doses of heparin at a total cost of £6,301. The average cost per patient for the change in heparin policy was therefore £161 per patient.

Table 2 details the number of thromboembolic and bleeding complications encountered over the two-year periods for all patients admitted with a hip fracture.

**Table 2.** Number of thromboembolic and bleeding complications over the two-year periods.

Year	2012	2013	*p* value
Number of patients	486	465	
Mean age [range]	81 [22–101]	81 [21–102]	
Female	339 (69.8)	319 (68.8%)	
Venous thrombosis	9 (1.9%)	5 (1.1%)	0.4
Pulmonary embolism	1 (0.2%)	0	1.0
Wound haematoma	1 (0.2%)	3 (0.6%)	0.36
Gastrointestinal bleeding	6 (1.2%)	11 (2.4%)	0.22
Mortality at 30 days	37 (7.6%)	27 (5.8%)	0.3

## Discussion

There remains continuing debate about the choice of thromboembolic prophylaxis after hip fracture surgery and elective joint arthroplasty. The most recent update to the American College of Chest Physicians guidelines in conjunction with the American Academy of Orthopaedic Surgeons, has considerably revised their previous recommendations on thromboembolic prophylaxis. In formulating the guidelines there has been a shift from basing the recommendations on the occurrence of asymptomatic thrombotic events, to symptomatic events. The level of evidence has been reassessed and has been downgraded from 1A to that of 1B. Current recommendations within these guidelines are now for either low-molecular weight heparin, aspirin, warfarin, rivaroxaban, dabigatran, apixaban or portable mechanical compression for a minimum of 14 days [3].

The initial Scottish SIGN guidelines recommended low dose aspirin for hip fracture patients, but for the most recent update this was changed to recommending low-molecular weight heparin for 28 days [5]. For England the most recent NICE guidelines are now at odds with those of the American College. NICE has graded the level of evidence on this topic as 1+ and stated that aspirin alone is insufficient prophylaxis [4]. This had led to many hospitals in England changing from using either low dose aspirin or a short course of heparin to an extended course of heparin. The NICE guidelines do consider cost effectiveness of thromboembolic prophylaxis, but in their calculations for hip fracture patients assumed that only 8% of patients would require a district nurse to administer injections at home after discharge from hospital. This figure was based on series of younger patients having knee arthroscopy [10]. The present study found that a third of hip fracture patients required a district nurse, thereby making the NICE calculation on cost-effectiveness unsound.

This study did not consider the costs of treating bleeding or thromboembolic complications. This study only considered the much larger costs incurred from the increased number of heparin doses and the district nurse costs to give a cautious estimate for the total costs incurred. The calculations used in this study were based on a fixed 28-day course of heparin, not a variable 28- to 35-day course as recommended in the NICE guidelines. Furthermore, the information on heparin use was unknown for nine patients and these patients were not included in the calculations of increased heparin use.

There are also a number of other potential costs involved with a prolonged course of heparin to consider that have not been included in this study. These include the nursing time for administration of extra heparin doses to inpatients, time taken by nursing staff to explain to the patient or relatives how to self-administer heparin, time taken by the medical staff to prescribe additional heparin, time taken by the pharmacist staff to supply additional doses of heparin, provision of written instruction on how to self-administer heparin, provision of sharps bins for the disposal of needles, services for the disposal of the sharps bins after use and the checking of platelet count and renal function whilst the patient is taking heparin. Many of these aforementioned costs are difficult to quantify, as they are included within normal working practices, or may be provided by the pharmaceutical company.

The number of reported thromboembolic and bleeding complications within this study is similar to that reported from other studies on hip fracture patients [7, 11]. The numbers from this study as detailed in Table 2, suggest a small decrease in thromboembolic complications and increase in the bleeding complications between the 2 years. With the limited patient numbers, it is unrealistic to be able to show a statistically significant difference between the two groups and a considerably larger number of patients would be required for this.

Based on an estimated number of 77,000 hip fractures occurring each year in the England [12], this study suggests the total cost for a change from a short course of heparin to a more prolonged course would be in excess of 12 million pounds. In this time of financial austerity in the health service there is a need to question the appropriateness of spending such large amounts of money on extended thromboembolic prophylaxis for hip fracture patients. Because of the lack of large independently conducted randomised trials on heparin it still remains unclear as to the relative merits and complications of heparin [2, 6]. This is not the case for aspirin for which both the benefits and complications have been assessed with randomised trials [13]. Until such studies are undertaken for heparin the cost effectiveness of heparin after hip fracture surgery remains unknown.

## Conflict of interest

The author has no conflict of interest with this study.
